# Orofacial Myofunctional Therapy to Treat Residual Obstructive Sleep Apnoea in Children Treated with Adenotonsillectomy and Maxillary Expansion: An Interventional Study

**DOI:** 10.3390/jcm15197676

**Published:** 2026-10-04

**Authors:** Derek Mahony, Niroj Bhattarai, Peter Petocz, Peter Borbély, Krisztina Kárpáti

**Affiliations:** 1 Department of Orthodontics and Pediatric Dentistry, Faculty of Dentistry, University of Szeged, Tisza Lajos Krt. 64–66., 6720 Szeged, Hungary; derek.mahony@fullfaceorthodontics.com.au (D.M.); drborbelypeter@gmail.com (P.B.); drkarpatik@gmail.com (K.K.); 2 Research Faculty, Excellence in Orthodontics and Dentofacial Orthopaedics (EODO), Haberfield, NSW 2045, Australia; 3 Graduate Research Academy, Macquarie University, Sydney, NSW 2109, Australia; peter.petocz@mq.edu.au

**Keywords:** paediatric, obstructive sleep apnoea (OSA), oral myofunctional therapy (OMFT), respiratory disturbance index (RDI), residual OSA, multidisciplinary treatment, polysomnography (PSG)

## Abstract

**Background:** In children with residual obstructive sleep apnoea (OSA) despite combined adenotonsillectomy (TA) and semi-rapid maxillary expansion (SRME), the optimal further management remains unclear. This study evaluated the effect of a 12-month programme of orofacial myofunctional therapy (OMFT) on residual OSA in this population. **Materials and Methods:** In this retrospective cohort study, 80 children (aged 7–9 years) with polysomnography-confirmed residual OSA (respiratory disturbance index [RDI] ≥ 1 event/h) after combined TA and SRME were followed for 12 months. Forty-one children completed a 12-month course of OMFT, and 39 did not, due to compliance concerns, cost/access barriers, or parental preference. RDI at 12-month follow-up (RDIfinal) was compared between groups by *t*-test and by a covariate-adjusted general linear model. **Results:** Mean baseline RDI did not differ between groups (*p* = 0.21). At follow-up, mean RDIfinal was 0.49 events/hour in the OMT group versus 5.26 in controls (*p* < 0.001; Cohen’s *d* = 3.64), a difference that persisted after adjustment for treatment order, sex, age, race, and BMI (*p* < 0.001). **Conclusions:** A 12-month OMT programme was associated with near-normal respiratory indices in children with residual OSA after combined TA and SRME, while untreated residual OSA showed mild deterioration. Given the non-randomised design, this should be interpreted as a strong association rather than a confirmed causal effect, and a randomised trial is warranted.

## 1. Introduction

Adenotonsillar hypertrophy is generally regarded as the principal cause of paediatric obstructive sleep apnoea (OSA) [1,2,3,4], and adenotonsillectomy (TA) has accordingly become the standard first-line intervention [5,6,7,8,9]. Despite surgery, residual OSA has been reported in 13–29% of children following TA. This figure rises to as much as 75% among those who are overweight or obese [10,11,12,13]. Persistent sleep fragmentation, and repeated arousal in childhood, have been linked to poorer school performance, irritability, and behavioural disturbance. Maxillary expansion, alone or in conjunction with TA, in patients with OSA and a narrow maxilla, has also been shown to decrease the severity of OSA. However, research confirms that it cannot fully resolve OSA [14,15]. In our own cohort, the combination of TA with semi-rapid maxillary expansion (SRME) achieved a substantial reduction in the respiratory disturbance index (RDI), yet a subset of children remained short of full normalisation [16]. It is this unresolved residual disease, present despite two structural interventions, that the present study sets out to address.

Part of the explanation may lie in the fact that OSA is rarely a purely anatomical problem [17]. Alongside the airway size, contributors such as poor pharyngeal muscle responsiveness, a low arousal threshold, and high loop gain have been increasingly implicated [18]. Treatments confined to anatomy, such as CPAP, surgery, and dental appliances, can consequently underperform when these non-anatomical traits predominate, particularly in milder disease [19,20]. TA and SRME, whatever their individual merits, each correct a single structural deficit—lymphoid bulk in one case, transverse maxillary width in the other—without addressing muscle tone or responsiveness in the pharynx itself [21]. Orofacial myofunctional therapy (OMFT) is directed precisely at this gap. Through isotonic and isometric exercise, it aims to build tone, endurance, and coordination in the pharyngeal and peripharyngeal musculature, encouraging the tongue to rest against the palate rather than fall posteriorly [22,23,24]. This favours nasal over oral breathing, and apparently sustains genioglossus-mediated dilator tone across sleep stages, including REM sleep, when tone would otherwise decline and the risk of collapse is greatest. Myofunctional assessment has identified measurably poorer tongue posture, tongue strength, lip strength, and soft-palate mobility in children with OSA, suggesting the significance of OMFT in this population [25,26].

The evidence base for OMFT itself is modest, but consistent. Guimarães and colleagues were the first to test oropharyngeal exercises formally, in a randomised trial of adults with moderate OSA [27], and a later meta-analysis by Camacho and colleagues, spanning adult and paediatric studies, reported apnoea–hypopnoea index (AHI) reductions of roughly 50% in adults and 62% in children, alongside improvements in snoring and daytime sleepiness [28]. The nearest paediatric precedent to the present design is a small retrospective cohort in which children took up OMFT, after adenotonsillectomy and orthodontic treatment, by referral and family preference, rather than randomisation—though that study looked at the prevention of relapse in children already cured, rather than the treatment of confirmed residual disease [29]. To our knowledge, no prior study has examined OMFT in children with confirmed residual OSA following combined adenotonsillectomy and maxillary expansion.

The present study evaluates the effectiveness of a twelve-month OMFT programme on residual OSA in prepubertal children who had already undergone combined TA and SRME, comparing respiratory outcomes at twelve months between those who completed the OMFT programme and those who did not.

## 2. Materials and Methods

### 2.1. Study Design

This retrospective cohort study was conducted as a 12-month follow-up analysis on the sample of patients previously described in a study [16] evaluating the effects of adenotonsillectomy (TA) and semi-rapid maxillary expansion (SRME), individually and in combination, on the respiratory disturbance index (RDI) in prepubertal children with obstructive sleep apnoea (OSA). The current study aimed to assess the impact of orofacial myofunctional therapy (OMFT) on residual OSA in children who had already completed both TA and SRME, regardless of the order in which those interventions were performed. Ethical approval was obtained from the Medical Ethics Committee of the University of Greater Manchester. Informed consent for the use of clinical data for research purposes was originally obtained from the parents or legal guardians of all participants at the time of clinical care, and this consent was individually reconfirmed with each family prior to inclusion of their child’s data in the present study. The study adhered to the principles outlined in the Declaration of Helsinki.

### 2.2. Sample Selection

This cohort of 80 children was drawn from 3671 children aged 7 to 9 years who presented for their first orthodontic consultation across nine private orthodontic clinics in Australia operated by the same specialist between February 2007 and December 2022 [16]. At initial consultation, all children were screened for sleep-disordered breathing using the BEARS questionnaire; children scoring ≥5 out of 9 were considered at elevated risk and referred to a sleep physician for polysomnographic evaluation, from which the diagnosis of OSA and its severity were confirmed.

All children in this cohort completed both TA and SRME and underwent Level 1 polysomnography (PSG) at baseline (Time 1), post-first intervention (Time 2), and post-both interventions (Time 3, RDI 3), and hence, were eligible for inclusion in this follow-up study. Patients were selected for the OMFT/control comparison based on the following criteria:

- Complete Level 1 in-laboratory PSG records at baseline, post-first intervention, post-both interventions (Time 3), and 12-month follow-up (Time 4).
- Confirmed residual OSA at Time 3, defined as RDI ≥ 1 event/h.
- Clear classification as either having completed a 12-month course of OMFT or as not having undertaken OMFT.
- Aged 7–9 years at original baseline, with no genetic syndromes, craniofacial abnormalities (beyond maxillary constriction), developmental issues, maxillofacial trauma, or tumour history.

Exclusion criteria mirrored the parent study, including incomplete records, home-based or non-Level 1 sleep studies, receipt of any additional ENT or orthodontic/orthopaedic airway intervention during the 12-month follow-up period, other than OMFT itself, partial or ambiguous OMFT engagement that could not be clearly classified, and withdrawal of parental/guardian consent.

**Figure 1:**
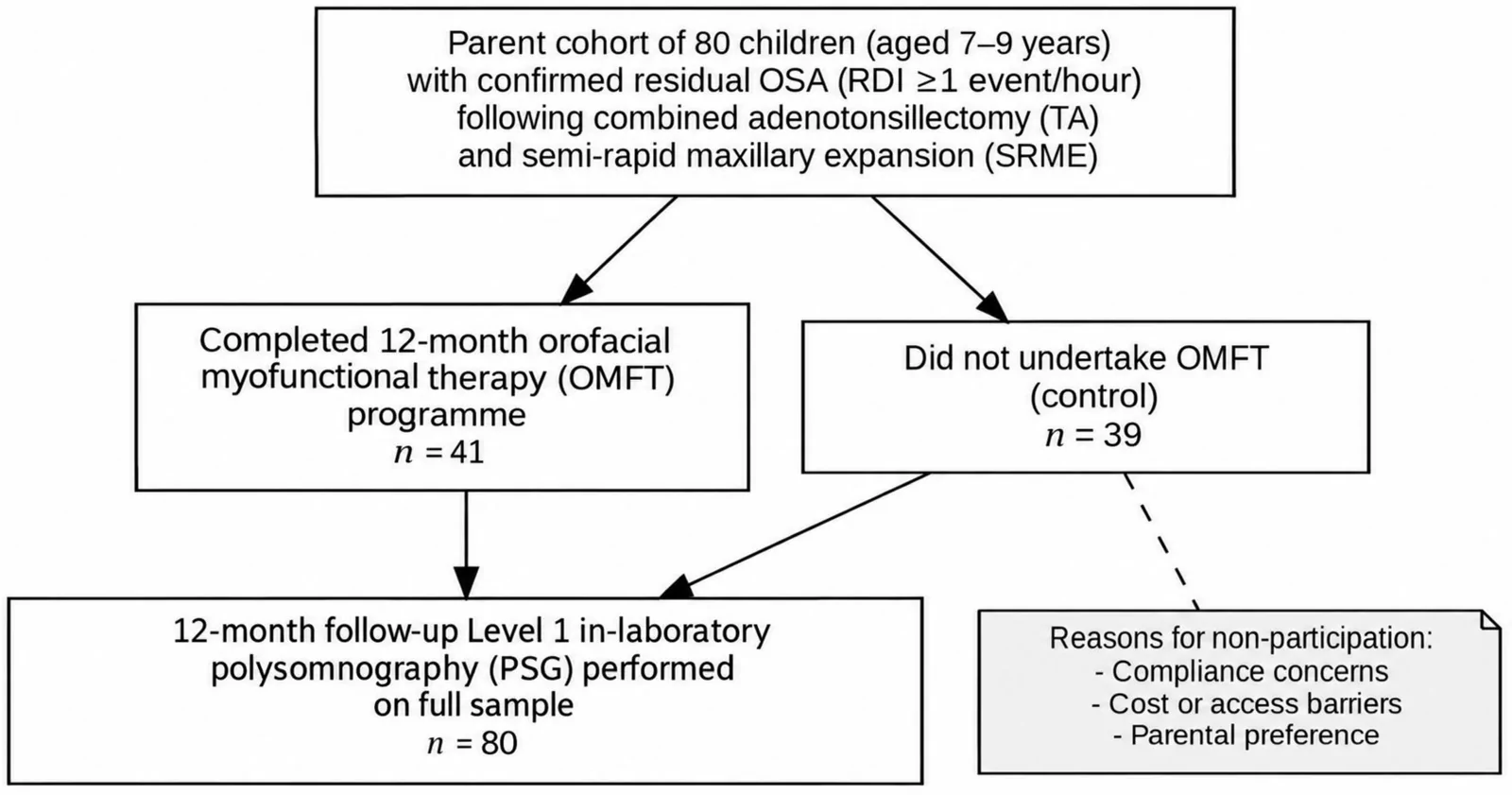
Patient selection flow diagram.

The sample was divided into two groups based on residual-OSA management, determined by family compliance capacity, cost/access, and parental preference, rather than randomisation:

1. **Group 1 (OMFT; *n* = 41)**: Children who completed a 12-month course of orofacial myofunctional therapy.
2. **Group 2 (Decline/Non-Compliant; *n* = 39)**: Children who did not undertake OMFT, due to compliance concerns, cost or access barriers, or parental refusal of any further treatment, for their child’s residual OSA (Figure 1).

Treatment allocation was not randomised but reflected real-world clinical decision-making, consistent with the parent study.

### 2.3. Interventions

1. **Orofacial myofunctional therapy (OMFT)**: OMFT was delivered by a qualified myofunctional therapist, over 12 months, comprising 24 clinical sessions held fortnightly, combined with 10–15 min of prescribed daily home exercises. Parents were required to complete a daily diary recording compliance with the home exercise programme. The myofunctional therapist reduced the frequency and/or duration of exercises once correct execution of the home programme had been confirmed, consistent with standard step-down myofunctional therapy protocols.
2. **Control (No Intervention)**: Children in the control group received no structured myofunctional intervention over the 12-month follow-up period for the reasons described in Section 2.2. No child in either group received any new ENT, or orthodontic/orthopaedic intervention, during this interval.

A follow-up PSG was performed on the full sample of 80 children approximately 12 months after the post-both-interventions PSG (Time 3) of the parent study.

### 2.4. Data Collection

Original baseline and Time 3 (new baseline for this study) data were derived from the parent study records, including polysomnographic (PSG) measurement, demographic details, BEARS questionnaire scores, and cephalometric classifications. At 12-month follow-up, PSG measurements, anthropometric data (height, weight, BMI category), and BEARS questionnaire scores were re-collected, together with OMFT session-attendance and home-practice diary data for Group 1.

BEARS questionnaire scores were used solely as a screening threshold to identify children requiring referral for polysomnography, as described in Section 2.1, and are reported here as a binary screen-positive/negative indicator rather than a continuous outcome measure. BMI was categorised into four ordered groups (underweight, healthy weight, overweight, obese) based on the treating clinician’s standard assessment at the time of care. For the covariate-adjusted analysis in Section 3.5, these categories were coded numerically and entered as an ordinal covariate.

#### Polysomnography (PSG) Measurements

All PSGs, including the 12-month follow-up study, were Level 1 in-laboratory studies conducted at accredited paediatric sleep centres, following Australasian Sleep Association guidelines. Respiratory events were manually scored by certified sleep technicians, blinded to group allocation. The primary outcome measure was the respiratory disturbance index (RDI), defined as the average number of apnoeas, hypopnoeas, and respiratory effort-related arousals (RERAs) per hour of sleep. Scoring criteria followed American Academy of Sleep Medicine (AASM) paediatric rules:

1. Apnoea: ≥90% reduction in airflow for ≥2 missed breaths, with continued respiratory effort (obstructive) or absent effort (central).
2. Hypopnoea: ≥30% reduction in airflow for ≥2 missed breaths, associated with ≥3% oxygen desaturation or arousal.
3. RERA: Sequence of breaths with increasing respiratory effort leading to arousal, without meeting apnoea/hypopnoea criteria.

RDI at 12-month follow-up (RDIfinal) and its change from Time 3 were calculated for each participant.

### 2.5. Statistical Analysis

All statistical analyses were performed in IBM SPSS Statistics for Windows, Version 31.0 (IBM Corp., Armonk, NY, USA), with significance set at *p* < 0.05. Means and standard deviations were calculated for RDIfinal and difference in the RDI (dRDIfin) in the OMFT and control groups. The two groups were compared using an independent-samples *t*-test, with Levene’s test applied to check the assumption of equal variances and a Welch-adjusted statistic reported where this was not met. Effect sizes are reported as Cohen’s *d*, Hedges’ correction, and Glass’s delta, each with a 95% confidence interval. Because allocation to OMT was not randomised, RDIfinal was further modelled using a general linear model (univariate ANCOVA), with MFT status, treatment order, sex, age group, and race entered as fixed factors, and baseline (Time 3) RDI and BMI entered as covariates.

## 3. Results

### 3.1. Sample Characteristics

The sample consisted of 80 children who had confirmed residual OSA (RDI ≥ 1 event/h) after completing both adenotonsillectomy (TA) and semi-rapid maxillary expansion (SRME). At Time 3 (baseline for this study), the point immediately after both interventions were completed, and before any decision about OMFT had been made, mean RDI across the whole sample was 4.45 ± 1.71 events/hour. Participants were divided into two groups according to whether they subsequently undertook and completed orofacial myofunctional therapy (OMFT): 41 children completed a 12-month course of OMFT, and 39 did not, due to compliance concerns, cost or access barriers, or parental preference not to pursue further treatment for the child’s residual OSA. Baseline demographic characteristics and Time 3 clinical status for each group are presented in Table 1.

### 3.2. RDI at 12-Month Follow-Up (RDIfinal)

**Figure 2:**
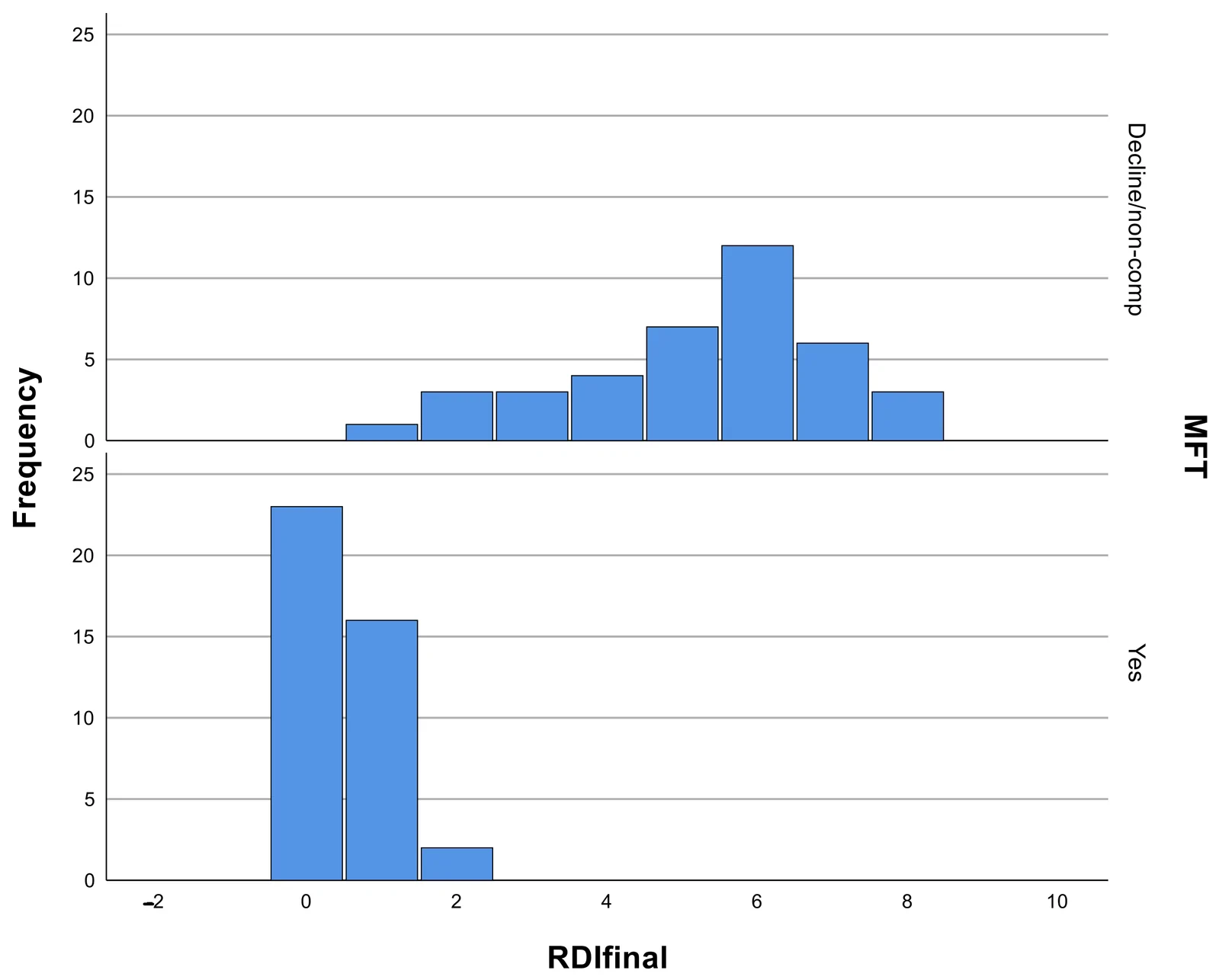
Distribution of respiratory disturbance index (RDI) at 12-month follow-up (RDIfinal), by group. (**Upper panel**): decline/non-compliant (control) group; (**lower panel**): OMFT group. Bars show the number of children (y-axis) at each RDIfinal value (x-axis, events/hour).

By the 12-month follow-up, the two groups had diverged substantially. Children who had completed OMFT showed a mean RDI of 0.49 ± 0.60 events/hour, a value within the normal range, whereas children in the control group remained at 5.26 ± 1.77 events/hour, still within the range of clinically significant residual OSA. This difference of 4.77 events/hour (95% CI 4.19–5.35) was highly significant (t(78) = 16.29, *p* < 0.001; Welch-adjusted t(46.12) = 15.96, *p* < 0.001), with a very large effect size across all three measures reported in Table 2b. Individual values ranged from 0 to 2 events/h in the OMFT group and from 1 to 8 events/hour in the control group, with no overlap between the upper end of the OMFT distribution and the lower end of the control distribution (Figure 2).

### 3.3. Change in RDI from Time 3 to Time 4 (dRDIfin)

**Figure 3:**
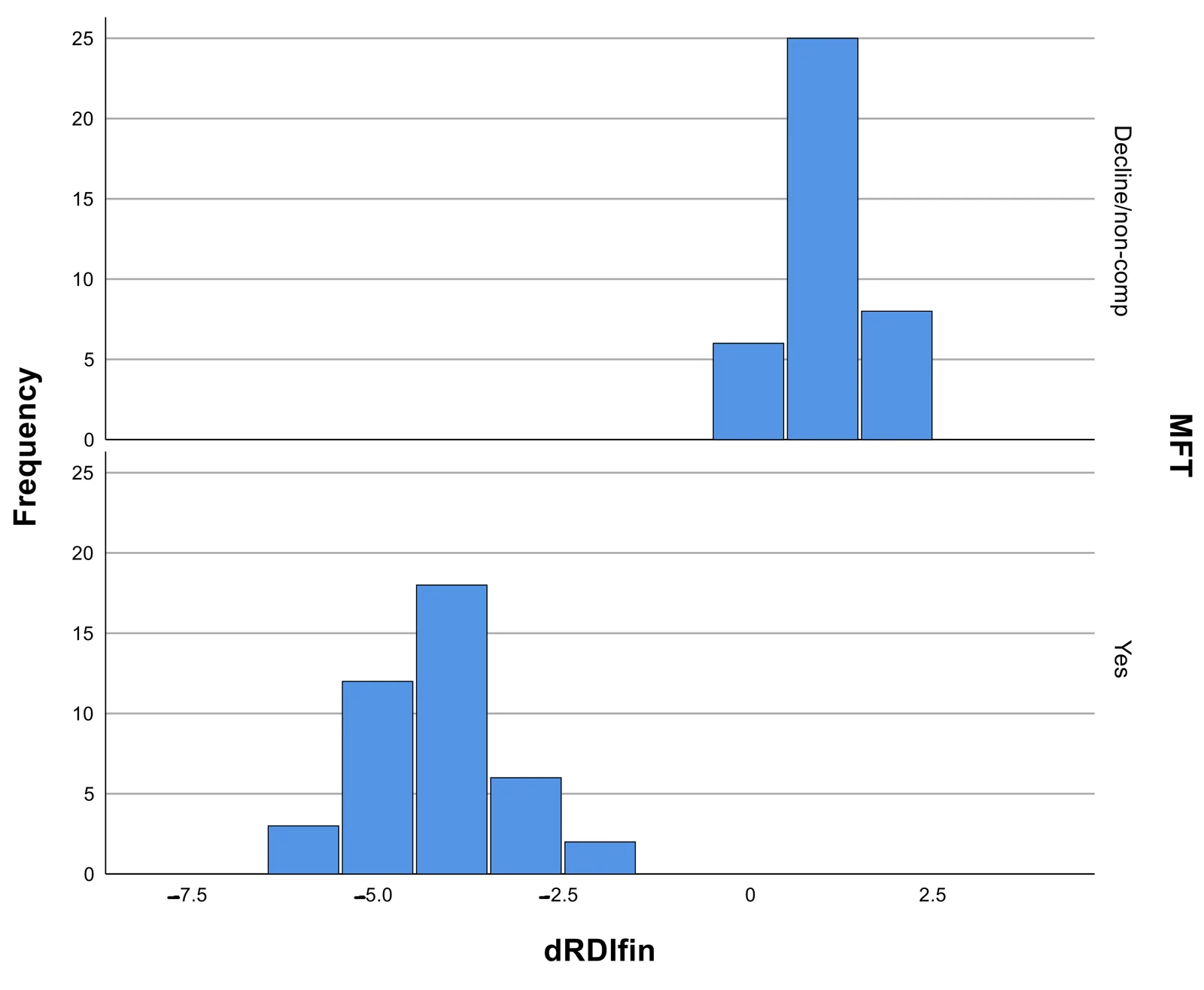
Distribution of change in RDI from Time 3 to 12-month follow-up (dRDIfin = RDIfinal − RDI3), by group. (**Upper panel**): decline/non-compliant (control) group; (**lower panel**): OMFT group. Negative values indicate improvement (RDI reduction); positive values indicate worsening.

Over the 12-month follow-up period, the OMFT group showed a mean reduction in RDI of 4.20 ± 0.95 events/hour, while the control group showed a mean increase of 1.05 ± 0.61 events/h. This between-group difference of 5.25 events/hour (95% CI 4.89–5.60) was highly significant (t(78) = 29.20, *p* < 0.001; Welch-adjusted: t(68.12) = 29.52, *p* < 0.001), with an exceptionally large effect size (Cohen’s *d* = 6.53, 95% CI 5.41–7.64; Hedges’ g = 6.47; Glass’s Δ = 5.50). Every child in the OMFT group showed improvement (dRDIfin ranging from −6 to −2 events/hour), while every child in the control group showed no change or worsening (dRDIfin ranging from 0 to +2 events/hour); the two distributions did not overlap (Figure 3).

### 3.4. Distribution of Outcomes

**Figure 4:**
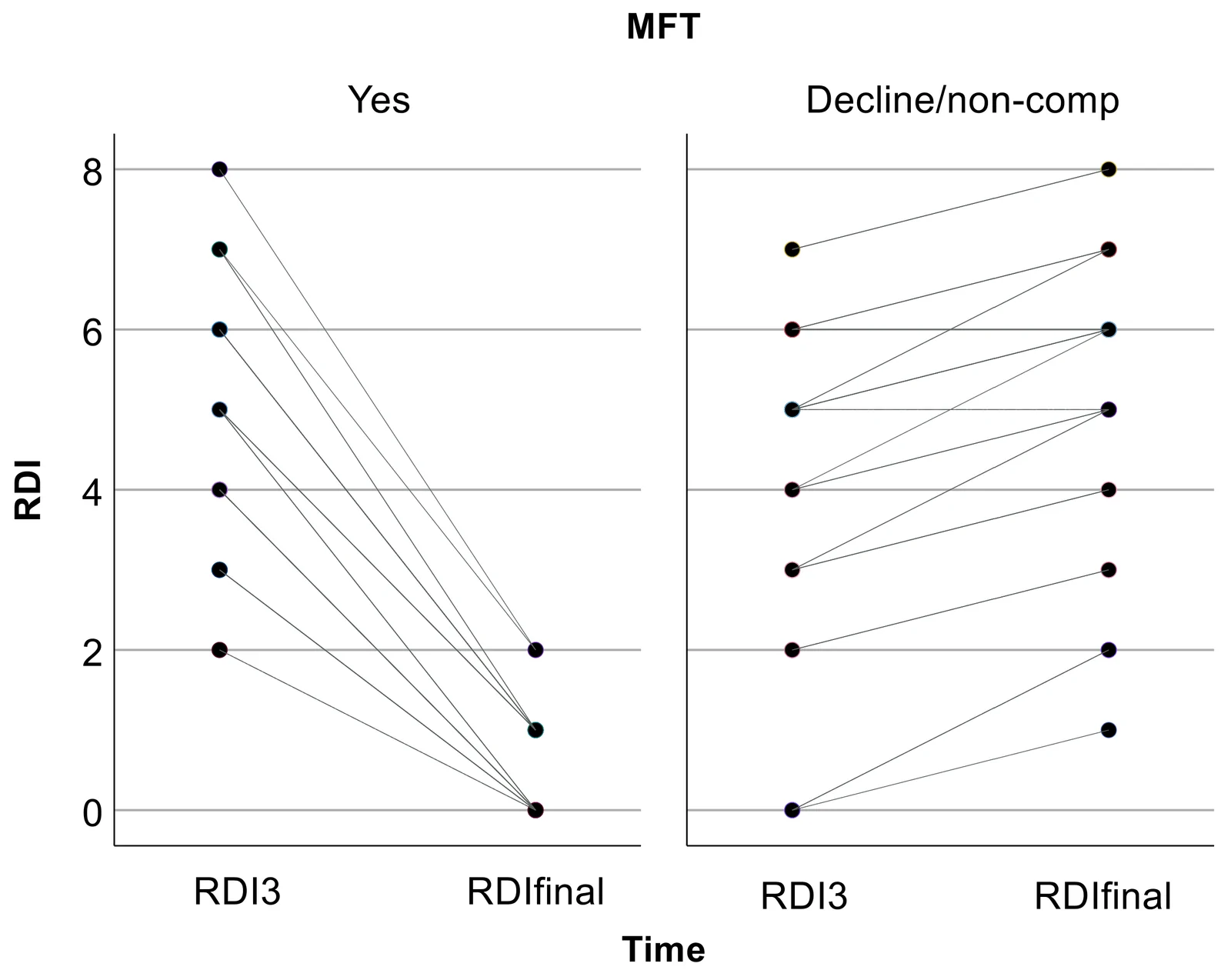
Individual RDI trajectories from Time 3 (RDI3) to 12-month follow-up (RDIfinal), by group. Each line represents one child. (**Left panel**): children who completed the OMFT programme; (**right panel**): children in the decline/non-compliant control group.

Examining each child’s individual trajectory, rather than their endpoint alone, yielded a consistent pattern. Over the 12-month follow-up period, mean RDI fell by 4.20 ± 0.95 events/hour in the OMFT group, while the control group’s mean RDI rose by 1.05 ± 0.61 events/hour, indicating that residual OSA left without further intervention did not remain stable, but tended to worsen modestly over the following year. The resulting between-group difference of 5.25 events/hour (95% CI 4.89–5.60) was again highly significant (t(78) = 29.20, *p* < 0.001; Welch-adjusted t(68.12) = 29.52, *p* < 0.001), with an exceptionally large effect size (Table 2b). Every child in the OMFT group showed improvement (dRDIfin ranging from −6 to −2 events/hour), while every child in the control group showed no change or deterioration (0 to +2 events/hour); the two distributions did not overlap at any point (Figure 4).

### 3.5. Covariate-Adjusted Analysis

A general linear model for RDIfinal in terms of MFT, adjusted for treatment order, sex, age group, race, BMI and baseline RDI (RDI3), found a significant effect of MFT (F(1,69) = 1492.99, *p* < 0.001), with baseline RDI also significant (F(1,69) = 133.72, *p* < 0.001) but no other significant factors or covariates (all *p* ≥ 0.135). The estimated marginal means (Table 3) show a large difference between MFT groups—0.28 for Yes versus 5.42 for Decline/non-comp—while the means for the other factors differ only marginally from one another. The parameter estimate for RDI3 was 0.41 (SE 0.08), reflecting the significant effect of baseline RDI on 12-month outcome. The overall model accounted for 96% of the variance in RDIfinal (R^2^ = 0.962, adjusted R^2^ = 0.956).

**Figure 5:**
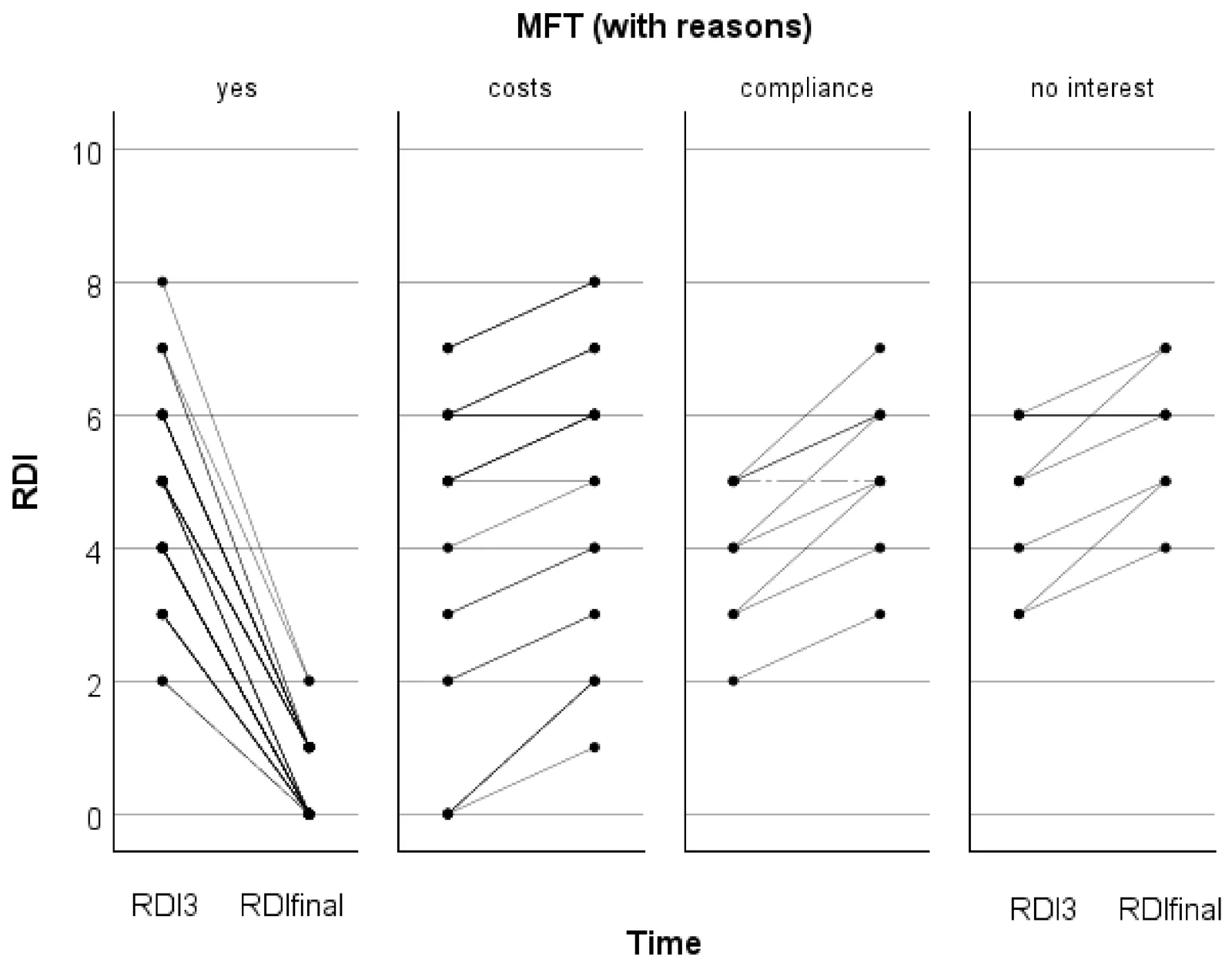
Individual RDI trajectories from Time 3 (RDI3) to 12-month follow-up (RDIfinal) with reasons for non-participation.

The reason for non-participation was available for the control group: cost/access barriers (*n* = 22), non-compliance (*n* = 9), and lack of interest (*n* = 8). Adjusted models showed the OMFT group differed significantly from all three subgroups on both outcomes, while the subgroups did not differ from one another (adjusted mean RDIfinal: 0.29 vs. 5.37–5.53; dRDIfin: −4.16 vs. 0.92–1.08), Given the small size of the non-compliance (*n* = 9) and lack-of-interest (*n* = 8) subgroups, this comparison was likely underpowered to detect moderate differences between reasons for non-participation, and the absence of a statistically significant difference should not be interpreted as evidence of true equivalence between these subgroups (Figure 5).

## 4. Discussion

This study found that children who completed the structured 12-month OMFT exercise showed a substantial and consistent improvement in their residual OSA having already undergone combined adenotonsillectomy and semi-rapid maxillary expansion. By 12 months, the OMFT group’s mean RDI had returned to within the normal range, whereas children who did not undertake OMFT remained in the residual-OSA range and, if anything, showed a small further rise in RDI over the same period. The two groups ended up with significant differences in individual children’s residual RDI as well as the amount of improvement seen, and they barely came close to each other. In fact, not one child, in either group, had a result that matched anyone in the other group.

The physiological plausibility of this finding is consistent with what is known about OMFT’s mechanism of action. Because TA and SRME each address a single anatomical contributor to obstruction, lymphoid bulk and transverse maxillary width, respectively, neither directly restores pharyngeal muscle tone or corrects habitual low-tongue posture, and mouth breathing. OMFT targets exactly this residual functional deficit, and the sustained increase in genioglossus-mediated dilator tone it is thought to produce, including during REM sleep when tone physiologically declines. This offers a coherent explanation for why children with anatomically adequate but functionally unresolved airways continued to improve under OMFT while untreated children did not.

The direction and general pattern of this result align with the limited existing paediatric literature. Bandyopadhyay et al.’s meta-analysis of ten paediatric studies found AHI fell from 4.32 to 2.48 events/hour, a 43% reduction [30]—notably, from a baseline close to the Time 3 RDI observed in this cohort (4.45)—making it a reasonably matched comparison by disease severity, even though the percentage effect is considerably smaller than the ~90% reduction seen in this study.

Treatment dose appears to be a recurring determinant of whether myofunctional therapy produces a measurable effect in children and adults alike. Huang et al.’s paediatric cohort found no significant change in AHI across the full treated group, but a significant reduction confined to the minority (10 of 54 children, 18.5%) who achieved full protocol compliance [31]—echoing findings in the adult literature, where a network meta-analysis found no significant pooled AHI benefit overall, except when daily practice reached 30 min or more for at least 3 months [32]. The present study’s 12-month duration, and diary-monitored compliance, sit at the high end of this dose spectrum relative to most published paediatric protocols, which may partly explain the magnitude of the effect observed, independent of the selection-bias question addressed below.

The closest randomised comparator is Villa et al.’s trial, which evaluated oropharyngeal exercises specifically in children with residual OSA after adenotonsillectomy [33]—the same clinical population addressed here, and, unlike the present study, genuinely randomised. Villa et al. found a significantly greater AHI reduction with exercises than with sham nasal washing (ΔAHI 58.0% vs. 6.96%, individual AHI falling from 4.87 to 1.84 in the treatment arm, *p* = 0.004, with no significant change in controls), alongside significant improvements in oral breathing, labial seal, and lip tone, confined to the treatment group [33]. This provides genuine randomised-trial support for the qualitative pattern observed here, though over a much shorter intervention (2 months) in a considerably younger population (mean age ~5–6 years, versus 8.3 years in this cohort).

An earlier, non-randomised precedent shares the present study’s central limitation more directly than Villa et al.’s trial. Guilleminault et al. followed 24 children with a normalised AHI after adenotonsillectomy and orthodontic treatment, who were referred for myofacial re-education; only 11 actually received it, while 13 did not, through family follow-through rather than assignment [29]. At follow-up, performed 22–50 months after treatment completion, the 11 treated children showed no recurrence of breathing abnormality (AHI 0.5 ± 0.4/h), while the 13 untreated children showed clinically significant recurrence (AHI 5.3 ± 1.5/h, *p* = 0.001), alongside significant differences in oxygen saturation and flow limitation. This pattern—a clean separation between a treated and an untreated group under non-randomised, family-driven allocation—closely mirrors the present study’s design and result, and is therefore better read as a shared methodological limitation than as independent confirmation of a true treatment effect, a point taken up further in Section 4 below.

Saccomanno et al.’s review of nine randomised trials across children and adults concluded that myofunctional therapy’s inconsistent clinical adoption reflects variable protocols, poorly quantified adherence, and short follow-up (typically 2–6 months) more than genuine doubt about its direction of effect [34]. The present study’s 12-month follow-up and diary-recorded compliance data address two of these three gaps directly, strengthening the interpretability of the result relative to much of the existing paediatric literature.

The finding that untreated residual OSA did not simply remain stable, but became mildly worse over 12 months, is itself noteworthy. It suggests that residual disease following combined surgical and orthodontic treatment should not be regarded as a fixed, tolerable endpoint requiring no further action, but as a state that may deteriorate in the absence of intervention, plausibly reflecting ongoing somatic growth, ongoing habitual mouth breathing, or gradual loss of whatever partial compensation existed at Time 3.

This study has several notable strengths: to our knowledge, it is the first to evaluate orofacial myofunctional therapy specifically in children with confirmed residual OSA following combined adenotonsillectomy and maxillary expansion, rather than in children at risk of relapse after apparent cure; the 12-month intervention period and structured, diary-recorded compliance monitoring exceed the duration and adherence documentation typical of prior studies in this field; all respiratory outcomes were derived from objective Level 1 in-laboratory polysomnography, scored by technicians blinded to group allocation; and the two comparison groups were well balanced at baseline on age, sex, and residual disease severity (Table 1), which strengthens confidence that the observed difference reflects more than a pre-existing disparity. Nonetheless, the principal limitation of this study is that allocation to OMFT was not randomised: families chose, or were guided toward, therapy based on compliance capacity, cost and access, and personal preference. A covariate-adjusted analysis (Section 3.5) confirmed that the association between OMFT and 12-month RDI persisted, with an almost identical magnitude, after adjustment for treatment order, sex, age group, race, BMI, and baseline RDI severity—indicating that none of these measured variables account for the observed difference. This strengthens confidence in the result but does not resolve it: adjustment can only rule out confounding by variables that were measured, and it remains possible that unmeasured family-level factors associated with the capacity to complete a demanding 12-month therapy programme—rather than OMFT itself—contributed to the difference observed. The reason for non-participation was analysed as a secondary check (Section 3.5), though given the small size of these subgroups this analysis was likely underpowered, and the finding of no significant difference between reasons should be interpreted with caution rather than as evidence that the specific reason for non-participation is irrelevant to the outcome.

While the baseline balance described above rules out imbalance on the variables measured, families who successfully completed 24 fortnightly sessions over 12 months, together with daily supervised home practice, may plausibly differ from families who did not in unmeasured ways—general health engagement, household stability, or capacity to sustain a demanding therapeutic commitment—any of which could independently influence a child’s respiratory trajectory. The completeness of the separation between groups is consistent with a genuine and substantial treatment effect, but it is also the pattern one would expect if allocation itself was tied to such unmeasured factors, and the present, unadjusted analysis cannot fully distinguish between these explanations. The narrow range of residual RDI values in this cohort may also have contributed to the completeness of this separation independent of selection effects: a treatment effect of the magnitude observed here, relative to a measurement range this compressed, would be expected to produce limited overlap between groups even under random allocation, since there is little room within the scale for treated and untreated trajectories to cross.

Further limitations include the single-centre, private-practice setting, which may limit generalisability to broader or less airway-conscious populations; the 12-month follow-up window, which, while longer than most prior studies in this field, remains short relative to the craniofacial and pubertal changes that may themselves influence airway dimensions over a longer horizon; and reliance on parent-completed compliance diaries, which are subject to reporting bias and were not independently verified.

## 5. Conclusions

This study found that a 12-month programme of orofacial myofunctional therapy was associated with a return to near-normal respiratory function in children with residual OSA after combined adenotonsillectomy and semi-rapid maxillary expansion, while untreated children remained in the residual-OSA range and showed mild worsening over the same period. This pattern was directionally consistent with the two most closely comparable prior studies, though the effect observed here was larger than typically reported. Because allocation to therapy was not randomised, and the non-randomised comparator in the literature shares this same limitation, the result should be interpreted as a strong association rather than proof of a causal effect. A randomised controlled trial with objective compliance monitoring and longer follow-up is warranted to confirm this finding.

## Figures and Tables

**Table 1:** Demographic characteristics.

Characteristic	OMFT (*n* = 41)	Control (*n* = 39)	*p*
**Mean age (years)**	8.34 ± 0.53	8.27 ± 0.63	0.64
**Sex: male/female**	18/23	16/22	0.58
**Time 3 RDI, mean ± SD (events/h)**	4.68 ± 1.39	4.21 ± 1.99	0.21

**Table 2:** (**a**). Between-group comparison of RDIfinal and dRDIfin. (**b**). Effect sizes.

(a)					
Outcome	OMFT (*n* = 41), Mean ± SD (SE)	Control (*n* = 39), Mean ± SD (SE)	Mean Difference (95% CI)	t (df)	*p*
**RDIfinal (events/h)**	0.49 ± 0.60 (0.09)	5.26 ± 1.77 (0.28)	4.77 (4.19–5.35)	16.29 (78)	<0.001
**dRDIfin (events/h)**	−4.20 ± 0.95 (0.15)	1.05 ± 0.61 (0.10)	5.25 (4.89–5.60)	29.20 (78)	<0.001
(**b**)					
**Outcome**	**Effect Size**		**Point Estimate**	**95% CI**	
**RDIfinal**	Cohen’s *d*		3.64	2.92–4.36	
	Hedges’ correction		3.61	2.89–4.32	
	Glass’s delta		7.99	6.19–9.79	
**dRDIfin**	Cohen’s *d*		6.53	5.41–7.64	
	Hedges’ correction		6.47	5.36–7.57	
	Glass’s delta		5.50	4.21–6.77	

**Table 3:** Estimated marginal means for RDIfinal from the covariate-adjusted general linear model, by MFT status and other model factors. Values are model-based (adjusted) means with standard errors and 95% confidence intervals, evaluated at the model’s covariate means (baseline RDI = 4.45, BMI = 2.04).

Factor	Value Label	Adjusted Mean RDIfinal	Std Error (SE)	95% CI
MFT	No	5.42	0.10	5.22–5.63
	Yes	0.28	0.11	0.07–0.49
Order	ENT-First	2.73	0.12	2.50–2.96
	Exp-First	2.98	0.11	2.75–3.20
Sex	Male	2.90	0.11	2.68–3.12
	Female	2.80	0.10	2.60–3.00
Age Group	7.0–7.9	3.03	0.13	2.77–3.28
	8.0–8.6	2.75	0.13	2.49–3.00
	8.7–9.0	2.78	0.11	2.56–3.00
Race	Caucasian	2.53	0.20	2.14–2.92
	East Asian	2.89	0.09	2.72–3.07
	South Asian	3.11	0.16	2.79–3.42
	Others	2.88	0.18	2.52–3.24

## Data Availability

The datasets generated and/or analysed during the current study are available from the corresponding author upon reasonable request.
